# Pathway-Based Drug-Repurposing Schemes in Cancer: The Role of Translational Bioinformatics

**DOI:** 10.3389/fonc.2020.605680

**Published:** 2021-01-14

**Authors:** Enrique Hernández-Lemus, Mireya Martínez-García

**Affiliations:** ^1^ Computational Genomics Division, National Institute of Genomic Medicine, Mexico City, Mexico; ^2^ Centro de Ciencias de la Complejidad, Universidad Nacional Autónoma de México, Mexico City, Mexico; ^3^ Sociomedical Research Unit, National Institute of Cardiology “Ignacio Chávez”, Mexico City, Mexico

**Keywords:** pathway-based methods, drug repurposing, translational bioinformatics, computational oncology, PharmaOncology

## Abstract

Cancer is a set of complex pathologies that has been recognized as a major public health problem worldwide for decades. A myriad of therapeutic strategies is indeed available. However, the wide variability in tumor physiology, response to therapy, added to multi-drug resistance poses enormous challenges in clinical oncology. The last years have witnessed a fast-paced development of novel experimental and translational approaches to therapeutics, that supplemented with computational and theoretical advances are opening promising avenues to cope with cancer defiances. At the core of these advances, there is a strong conceptual shift from gene-centric emphasis on driver mutations in specific oncogenes and tumor suppressors—let us call that the silver bullet approach to cancer therapeutics—to a systemic, semi-mechanistic approach based on pathway perturbations and global molecular and physiological regulatory patterns—we will call this the shrapnel approach. The silver bullet approach is still the best one to follow when clonal mutations in driver genes are present in the patient, and when there are targeted therapies to tackle those. Unfortunately, due to the heterogeneous nature of tumors this is not the common case. The wide molecular variability in the mutational level often is reduced to a much smaller set of pathway-based dysfunctions as evidenced by the well-known hallmarks of cancer. In such cases “shrapnel gunshots” may become more effective than “silver bullets”. Here, we will briefly present both approaches and will abound on the discussion on the state of the art of pathway-based therapeutic designs from a translational bioinformatics and computational oncology perspective. Further development of these approaches depends on building collaborative, multidisciplinary teams to resort to the expertise of clinical oncologists, oncological surgeons, and molecular oncologists, but also of cancer cell biologists and pharmacologists, as well as bioinformaticians, computational biologists and data scientists. These teams will be capable of engaging on a cycle of analyzing high-throughput experiments, mining databases, researching on clinical data, validating the findings, and improving clinical outcomes for the benefits of the oncological patients.

## Introduction

Drug development is perhaps one of the most complex and challenging endeavors in biomedical science. Aside from the already daunting complexities behind pharmacological drug designs, there are also enormous difficulties derived from clinical, regulatory, intellectual property and commercial issues. Such a challenging environment has caused drug development to be a really slow and uncertain process. In the search for alternatives to treat the patients suffering from diseases such as cancer, researchers and clinicians have turned the attention to drug repurposing strategies. There are several advantages in the use of repositioning schemes for already existing validated, toxicologically safe and—no less-important—regulated pharmaceuticals to treat neoplasms. This is, however, a route not devoid of its own challenges and caveats. To cope with molecular heterogeneity (in particular mutational variances) a shift has recently made to resort to pathway-centered strategies that are aimed to approach the endeavor of drug repurposing armed with semi-mechanistic understanding of the mechanisms of action of the repurposed drugs on its new applications.

A number of successful approaches in this regard rely on the integration of methods from translational bioinformatics to face cancer data analysis with a clinician’s perspective in mind; computational intelligence to diminish biases both individual and methodological and systems biology to think in terms of processes and organisms aside from molecular cues. Only by effectively combining such theoretical approaches with improved clinical diagnostics and out of the box thinking, we will be able to live up to the promise of personalized oncology. Such endeavors will be particularly relevant for the treatment of tumors with scarce therapeutic options and those prone to develop resistance to therapy.

The rest of this work will be organized as follows: the following section will discuss the essentials of pathway-based drug repurposing methods. In particular, we will elaborate on how these methods are situated in relation to *de novo* drug designs, and what is the role played by advances in pharmaceutical informatics and personalized medicine. We will further describe the commonalities and differences of pathway-based repurposing and mutation centered approaches, by contrasting the strengths and limitations of both strategies. The following section is a discussion of recent advances in the field, including novel computational tools, a growing emphasis on the impact of these strategies in the clinical outcomes and the role of artificial intelligence and machine learning in drug repurposing approaches in cancer. We will also discuss on the development of novel omic approaches to probe tumors, the important role of drug delivery and precision drug targeting for repurposing, and recent advances in functional proteomics relevant to drug repositioning. Finally, some brief concluding remarks are outlined.

We will now pay attention to the importance of drug repurposing schemes as compared to *de novo* drug design, as this will guide the rest of our discussion of pathway-based approaches to anti-cancer therapy.

## Pathway-Based Drug Repurposing

### Drug Repurposing Versus *De* N*ovo* Design

Developing new anti-cancer drugs is of course a very important endeavor in itself. However, its timeline and route-maps are often very slow and costly. It is thus desirable that, in parallel with the synthesis and design of new anti-cancer compounds and their therapeutic combinations, we also consider strategies for the repurposing of the large number of already approved drugs (both anti-cancer and non-anti-cancer labelled) that may target known or soon-to-be-discover cancer players. Drug-repurposing has been considered as a good cost-effective strategy in order to widen-out the catalog of therapeutic options in oncology. A strategy that, in addition to be better suited to tackle better with molecular heterogeneity, is cheaper and faster to escalate to preclinical, clinical and tier studies stages, even up to clinical trials (1). In the case of approved drugs with known pharmacological interactions this may even pave the way to the development of tailor-made drug cocktails based on pathway-founded personalized medicine studies.

The latter point gains relevance in the light of a large body of evidence on the fact that combination therapies may lead to more powerful and effective results. In particular for the treatment of late-stage neoplastic tumors than single or sequential drugs combinations, given the large inter and intratumoral population heterogeneity (2, 3).

Of course, this is not to say that individualized, tailor-made polypharmacy therapy is free of caveats. Of notable relevance is the obvious fact that repurposing schemes did not follow the development and testing procedures that the pharmaceutical industry often impose on their new products, regarding dosage, tissue specificity and so on, and the fact that repurposed drugs were not designed with multi-therapy in mind (4).

Aside from these fundamental limitations there are other challenges to systematic approaches to drug repurposing for anti-cancer therapy. There are also defiances of a methodological and multi-disciplinary nature: the rational design of multi-drug repurposing schemes is a daunting task requiring the collaboration of clinical oncologists and cancer biologists with computational biologists, bioinformaticians and even experts in artificial intelligence, to name but a few disciplines. In this regard, oncologist and pharmaceutical officers need to adapt current practices to benefit from the input of professionals trained to manage the enormous wealth of information on chemical, pharmacological and genomic databases. Also, the use of biomedical informatics specialists to analyze electronic health records of the patients subjected to certain treatments. Let us consider some of these instances in more detail.

### Pharmaceutical Chemo-Informatics in Cancer Therapy

One relevant application of high level computational analysis is the use of data mining and computational intelligence for drug chemo-informatics, or pharmo-informatics. Particularly relevant for repurposing schemes is *off-target analysis*. The vast majority of drugs and other compounds used in pharmacological therapy have a large number of off-target effects (OTEs), i.e., additional targets or mechanisms aside from the main (intended) therapeutic mechanism of action (MoA). OTEs are often the actual basis of a large number of drug repurposing strategies. Due to combinatorially large “search spaces”, consequence of the systemic nature of MoAs, looking at OTEs is an endeavor that is difficult (and extremely slow) to perform by humans alone. Computationally assisted interrogations of the very large datasets currently available on drugs, its targets and its MoAs, allow for a sped-up process—often by narrowing down the available options—allowing the clinician to select from a handful alternatives and not from among thousands of them (5, 6).

Additional computer-aided methods of drug-repurposing include the hybrid use of knowledge discovery in databases (KDD) and molecular profiling/modelization to search for novel drug-target interactions. The use of machine learning and other computational and statistical intelligence techniques to screen the huge molecular catalogues, searching for drug-target interactions is gaining a lot of attention. By combining KDD and machine learning with high-throughput *in vitro* assay screening (HTS) it has been possible to devise efficient therapeutic strategies to treat multifactorial diseases such as cancer, largely outperforming single-drug approaches (7).

Interestingly, not only mono-therapeutic drug target interactions need to be considered in these designs. The relevant issues of molecular and phenotypic heterogeneity in cancer tumors need to be taken into account to reach clinically-worthy anti-cancer therapeutic interventions, such as the case of targeted immunotherapy (8). Immunotherapy has gained a lot of attention recently. However, although a number of patients respond quite successfully, a large fraction does not share such benefits. This is very likely associated with the fact that there are important effects linked to the immunosuppressive nature of the particular tumor microenvironments. In such situations, it may be advisable to resort to personalized designs centered on the individually-perturbed metabolic and signaling pathways. The recent work by Li and collaborators considered how metabolic circuits are able to regulate intrinsic tumor-suppressing immunity pathways. A relevant number of these interactions have made their way onto the clinical trial stage (see, Table 1 in 8). Systematic repurposing of immunomodulatory drugs like thalidomide, lenalidomide and pomalidomide has been validated and supported by comprehensive assessment studies (e.g., QSAR) of computationally predicted biomarkers in patient-diverse cohorts (9).

The clinical oncology community remains skeptical, since the pharmacological efficacy of such treatments is still quite heterogeneous (10). One avenue to overcome skepticism (and to level up such variability) is the inclusion of immunotherapeutic drugs in polypharmacological designs. This strategy has been deeply discussed by Shen and collaborators, regarding the use of thalidomide as a drug to increase delivery and therapeutic efficacy of cis-platin (11). Thalidomide and its derivative compounds, however, are still subject of scrutiny (both as mono-drugs and in combination therapy) due to a series of reports of adverse side effects, including neurotoxicity (12) and teratogenic events (13).

### Patient-Centric Drug Repurposing

Aside from molecular mechanisms and off-target effects, drug-repurposing schemes face additional demands related to individual heterogeneity. These challenges start with the availability of optimal diagnostic tools that consider factors helping to stratify such heterogeneous response to therapy. This is yet another instance in which computationally-assisted methodologies (CAMs) and AI may prove useful (14–17). Aside from CAMs/AI, modelization approaches based on systems biology frameworks would permit improved phenotyping and prognostics, leading to better-suited drug repurposing strategies (18, 19). Computational studies, relying on patient-wise genomic information, are becoming an invaluable tool to study the influence of genetic alterations in tumor progression and cell survival. This information, in turn, is fundamental to unveil tumor-specific weaknesses pointing out to clues for the development of optimal constrained sets of targeted therapeutic interventions, including drug repurposing designs (20–22).

Drug repurposing schemes extend far beyond designing drug lists or drug-cocktails. Additional consideration has to be given to making proper regimes available to the patient (1, 23). The first one of such considerations deals with the establishment of appropriate dosage to achieve anti-cancer pharmacological activity, which in general may be quite different from the dosage intended for the original use of the repurposed drugs. Computational tools have been actually developed to solve this issue (24–26). There are other non-technical (or better, not biological) issues to take into account. One of them is related to intellectual property, in particular on how to deal with patent and licensing issues, both in the case of generic and proprietary treatments. There are also economic challenges to be overcome, taking into account that cancer-related clinical trials are often more expensive, need longer follow-ups and are very prone to failure than those of non-cancer drugs. Pharmaceutical companies may find the endeavor of conducting repurposing trials to be financially unworthy. Those latter issues, although relevant, are out of the scope of the present work and hence will not be further discussed, the interested reader may refer, for instance to (5) and references therein. Coming back to the drug-repurposing molecular studies issue, we will further discuss some aspects of translational bioinformatics strategies to improve the design of personalized, pathway-based anticancer drug repurposing schemes. We will start by considering mutation-targeted therapy as this was the beginning of anti-cancer treatments beyond the use of broad cytotoxic agents.

### Mutation-Specific Therapies as an Approach to Personalized Medicine in Cancer: Pros and Cons of Silver Bullets

Since the discovery of the first cancer-associated mutations and oncogenes, one central goal of anti-cancer therapy was that of looking for *cancer-causing mutations* (in particular tumor-drivers), to later resort to a tailor-armed approach to the molecular structure of *silver-bullets*, i.e., drugs able target tumors on an extremely specific fashion, while having no significant effects on non-tumor cells, often by targeting tumor-specific mutations.

**Figure 1:**
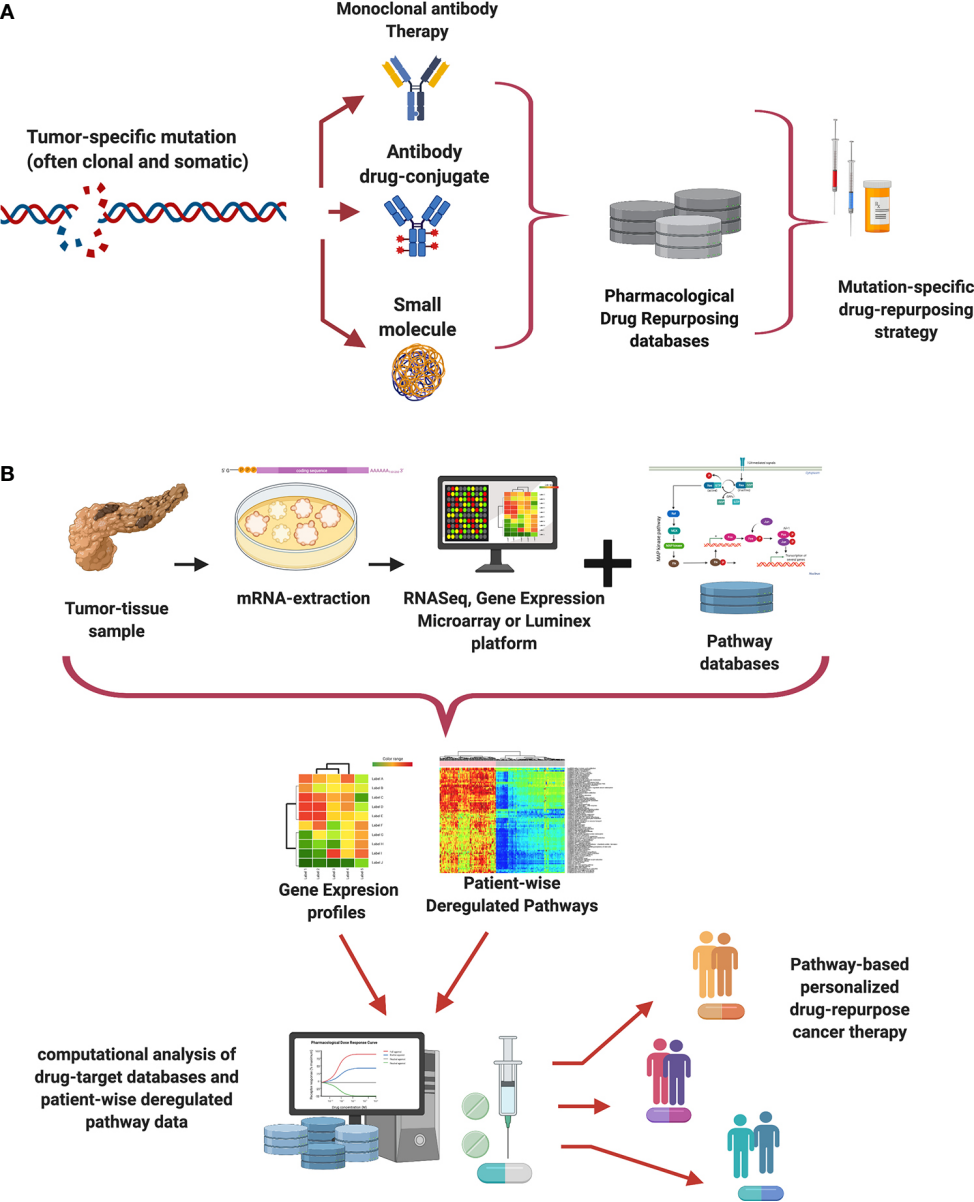
Mutation-specific and pathway-centric approaches to drug re-purposing. Panel **(A)** exemplifies a simplified workflow for drug repurposing based on tumor mutation profiling, Panel **(B)** shows one possible pipeline for drug repurposing based on pathway activities as proxied by gene expression analysis.

In **Figure 1A**, we present a schematic workflow for mutation profiling design of personalized anti-cancer drug repurposing. High-precision DNA sequencing is used to find a tumor specific mutation in a patient’s genome. If this mutation is annotated in a “cancer-panel”, the clinician will gain knowledge that may allow (specially if such a mutation is absent in the germline genome or in the healthy tissue) the search of a targeted therapy. Therapeutic alternatives may include monoclonal antibodies able to recognize the effect of the mutation at the protein level (27–29), composing an antibody-drug conjugate complex (30–33) or synthesizing a small molecule drug (34, 35). Armed with this knowledge, it is possible to look up into pharmacological databases, finding related drugs, along with off-targets and side effects (36–43). Those drugs are the long-sought *silver bullets*.

Unfortunately, with a few exceptional cases of highly penetrant mutations; most cancer patients have not benefited from these approaches (44, 45). Due to tumor mutational heterogeneity, most cancer mutations are rare, subclonal, often not causal and hence poorly annotated. The sequencing of more and more tumors, in combination with strong efforts to annotate the new variants may change this over time. However, things are not changing fast. A large scale study on the benefits of genome-driven oncology, the MOSCATO study (46, 47) concluded that purely genomic searches for cancer therapy are able to improve clinical outcomes in the minority of patients who undergo molecular screening. These results have diminished the emphasis on mutation-centered drug designs (48, 49). Mutational heterogeneity is fundamental to understand the challenges of mutation-centric studies. In recent times, mutational tumor variability has been unveiled at an unprecedented scale (50). Furthermore, pharmacologically-induced mutation is known to increase the malignancy and therapeutic-resistance (51).

The mutation frequency of well-known driver genes in metastatic breast cancer, for instance, has increased as a consequence of previous pharmacological treatment (52, 53). In this regard, the APOBEC family of APO enzymes, for instance, is known to be relevant for mutational heterogeneity (54, 55). These facts have led the pharmaco-oncology and clinical oncology experts to look up for alternative ways to face cancer therapeutics and drug repurposing. One of these avenues that is gaining a lot of momentum recently is that of pathway-based designs.

### Combining Pathway Analysis, Network Approaches, and Data Mining: the Shrapnel Approach

Alternatives to mutation-based therapeutic design exist and are becoming relevant. This is the case of studies based on functional pathway analyses based on gene expression profiling. One of these approaches combines pathway enrichment (56), pathway crosstalk (57) with the so-called *pathway deregulation analysis* (58) and network strategies (59) including probabilistic modeling and knowledge discovery in databases (60).

**Figure 1B** presents a simplified view of a pathway-based drug-repurposing workflow. Since it is known that gene expression, although quite heterogeneous, is better aimed at capturing functional similarities at the pathway level than mutational profiling. Such methods are transcriptome-based designs instead of a genome-based. The workflow starts by taking a tumor-biopsy sample from one patient. mRNA is extracted and purified from the sample. Then gene expression levels of the sample are measured either by RNA-Sequencing or by other technologies such as expression arrays, or a Luminex panel (7).

The rationale behind such pathway based methods has to do with a systems biology view on how to cope with the emergence of complex phenotypes (say tumors and tumor responses to therapy) from a myriad of (sometimes unknown) biomolecular interactions, metabolic reactions and signaling events. In the cases when the emergence of the phenotype is largely determined by one (or a handful) mutation events, genomic-variant centered approaches have proven quite efficient. However, more often than not, the emergence of the tumorigenic and tumor response to drug phenotypes is due to the interplay of a number (perhaps large) of mutually intertwined biological processes. Pathway based approaches to drug repurposing are intended to deal with such cases.

The gene expression sample profile is analyzed in the context of this large data corpus (sometimes by clustering or subtyping it), the next step consists in database mining from pathway databases such as KEGG (61), Reactome (62, 63), and Pathway Commons (64). One may either look up for a specific set of pathways (metabolic or immune system, for instance) or consider all currently annotated pathways. Once the set of pathways has been selected, it is possible to interrogate the databases looking for pathway-targeting drugs, this is molecules targeting key genes in the deregulated pathways.

Pathway deregulation metrics will allow for further filtering *via* joint analysis of pathway deregulation, differential gene expression, drug-target interactions, off-target, and side effects databases such as PharmGKB (65, 66), DrugBank (67), the Therapeutic Target Database, TTD (68) and others. Once these steps have been followed, we end up with a list of suggested therapies mapping the abnormal pathways linked to cancer in the different patients. These prioritized lists are the starting point of the work of the clinical oncologists and pharmaco-oncologists, as such, they are intended as mere tools, which, however useful, complement but do not replace the expertise of the clinical oncologist.

This workflow belongs to a more general family of pathway-based methods for individualized anticancer drug repurposing. As is known, biological functions are often represented as an interaction network of molecules within the cells. Such interactions are often captured in semi-mechanistic terms as pathways to try to capture the plethora of higher order biological functions (61). As we have said, often pathway-based strategies are founded on gene expression and other molecular profiling studies. Let us review some general ideas in this regard.

#### Gene Expression and Other Means of Molecular Profiling

One important challenge for the development of personalized drug repurposing approaches of anticancer therapy is molecular and phenotypic heterogeneity of the tumors. To tackle such variability, large scale databases like The Cancer Genome Atlas—Genomic Data Commons— (69–72) and others (73, 74), allow for analyses helpful to discern the commonalities and differences in gene expression features and associate them with the phenotypes and survival in thousands of cancer patients. Such systematic, data-driven studies, in turn, opened-up the possibility to create dynamic maps of tumor features and vulnerabilities by classes. Using these maps such as the CMAP led to the discovery of vulnerability biomarkers to guide clinical interventions (75).

Computational biology and AI studies of these huge omic databases along with clinical, data driven translational applications, are significantly improving patient-specific diagnostics and prognostics (76, 77). These, in turn, paved the way to enhanced designs to cancer therapeutics (78). Such large computational endeavors have also increased the success of targeted assays to determine the efficacy of competing therapies such as chemotherapy and hormone-guided designs (79) or the effects of combinatorial immune therapies (8).

#### Pathway Activity Profiling

Moving on from gene expression profiling to actual biological function is a daunting, unfinished task. However, a common approximation is given by analyzing which molecular pathways are deregulated, i.e., their activity functions in abnormal ways. Perhaps, the optimal experimental way to do this would be by resorting to massive phospho-proteomic and metabolomic experiments. However, technical and logistic challenges for accuracy and reproducibility of current proteomic technologies have discouraged further studies along these lines for the present moment. Hence, gene expression profiling has become the standard proxy used in large cohort studies of oncogenic pathway activity.

#### From Deregulated Pathways to Repurposed Drugs

After analyzing the individual repertoire of dysfunctional pathways (as proxied by the expression of key genes within them), it has been possible to devise pathway-centric approximations to drug repurposing. Let us discuss some remarkable cases. The case of breast neoplasms with challenging phenotypes is quite illustrative. A recent study led to the identification of nine breast tumor subtypes (instead of the usual 4 or 5 considered in the PAM50 classification). One of these subtypes, that went unobserved until this study, comprising about 7% of the cases (on a cohort of around 2000 tumors and 144 controls) resulted deregulated for 38 PKA pathways (80).

The importance of this finding for the therapeutic options to treat these tumors lies in the fact that despite being many protein kinase-driven pathways of great phenotypic impact, most of these pathways are all inducible by a single molecule: PRKACB which is a druggable gene. PRKACB is a target for Staurosporine, a p-glycoprotein/abcb1 inhibitor. Staurosporine induces cell death in (Luminal A-associated) MCF7 human breast cancer cells (81), and is known to also disrupt HUNK, a cell cycle-associated kinase in Her2+ tumors (82). In this way, Staurosporine is able to treat two different breast cancer subtypes (luminal and Her2+) by disparate yet related mechanisms that inhibit proliferation *via* PKA pathways. The same large scale study identified 9 EGFR-related pathways which can be targeted by FDA-approved drugs such as Anlotinib (83, 84). Anlotinib main use in cancer was already established to treat aggressively, drug-resistant tumors such as glioblastoma (85); Poziotinib (86–88). Other available EGFR-targeting molecules include Dacomitinib (89) and cationic polyamidoamine dendrimers (90).

Due to the binding nature of EGFR control, EGFR-modulation can also be attained by using glucocorticoids (91). However, hormone-mediated mechanisms of action are often less specific than other EGFR modulators mentioned, so caution must be taken (92, 93). We must notice that EGFR-centered therapies have resulted to be less effective than initially expected due to kinase repertoire heterogeneity. However, EGFR-targeting may result useful in combination therapy, for instance, to increase chemosensitivity in triple negative breast tumors. The mechanism proposed for this enhanced chemoselectivity is *via* reprogramming apoptotic signaling networks (94). The variability in response to EGFR-targeting is useful to introduce additional issues to be considered in the design of repurposing strategies. Two quite relevant among these issues are the effects of active pathway crosstalk and the role of secondary targets, in particular in relation to pharmacological resistance.

### Coping With Pharmacological Resistance: The Role of Pathway Crosstalk and Secondary Targets

A final, yet extremely relevant, issue to be considered in the design of pathway-based, individualized cancer therapy is the fact that the clinical efficacy of a drug goes well beyond the (static) molecular portrait given by the action of the drug on the pathway or pathways under consideration. The dynamic nature of drug activity depends on its effect, at the level of systemic, even organismal perturbations. Such phenomena occur within a densely interconnected signal transduction and metabolic network (95, 96). Given this, one must consider the MoA not only within the single instance of the prioritized pathways, but also in the context of all other biological phenomena occurring on their close surroundings (i.e., in the pathways’ network neighborhood). The phenomenon of pathway crosstalk, for instance, it is known to exert important effects on the onset and progression of pharmacological resistance (57, 97). Of course, pathway crosstalk has gone beyond network connectivity since, as stated, it is a highly dynamic process. For the cases in which one is able to anticipate crosstalk phenomena that may result relevant to pharmacological efficacy this must be considered in the initial design. At least dosage and coadjuvant therapies to prevent or diminish its effects must be analyzed in advance (98–100).

To date, a number of bioinformatic and computational biology resources have been developed to cope with the issue of pathway crosstalk in the context of drug repurposing (101–103). A recently proposed strategy is the use of crosstalk inhibition studies (104–107). However, other approaches include the evaluation of drug synergism (108–111), as well as cohort studies to evaluate and categorize crosstalk induced resistance (57, 112–114).

Aside from pathway crosstalk phenomena, in which the activity of several interconnected pathways is cross-regulated, there is also the issue of secondary molecular (and/or functional) targets. A secondary target of a drug has been defined as any target (a gene, protein, metabolite, etc.) whose associated MoA or downstream effects are not in line with the intended therapeutic mechanisms (115–117). Secondary target studies have been carried out for a long time. However, the availability of comprehensive database resources for high throughput assessment of secondary targets is relatively recent (118, 119).

Among the more relevant resources in the context of anti-cancer therapeutics, we can mention, for instance, the one maintained by the COSMIC consortium drug resistance database (CCDRD) (https://cancer.sanger.ac.uk/cosmic/drug_resistance (120, 121). CCDRD is indeed a quite comprehensive catalog of drug resistance events in cancer that is, however, limited in that it only considers somatic mutations. As we have already discussed, somatic-mutation therapy provides only a narrow window for therapeutic advances limited by the mutational heterogeneity of the tumors. Other approaches, although based on less comprehensive resources are also being considered (122). An outstanding example of its applications is the case of pembrolizumab (Keytruda) which is an immune checkpoint inhibitor drug. After looking up for secondary targets of pembrolizumab, Dang and coworkers found that some of them actually provide synergistic therapeutic effects (123).

## Discussion

### Recent Advances

Aside from the established computational frameworks for oncological drug repurposing already discussed, there is also a series of nascent, promising strategies that may complement them. Machine learning (ML) studies, for instance, are providing means of discovery relying more on the increasing abundance of omic and clinical data than on a deep knowledge of cancer biology (which is the case for most of the approaches already presented). The recent work of Issa and collaborators (124) summarizes well recent ML applications. Of noteworthy attention is the fact that some computational learning algorithms are already being applied beyond genomic and transcriptomic data. The role machine learning (Random forests, support vector machines, LASSO optimization) for ligand-based and docking studies (125, 126) for instance, has already resulted in therapeutic advances for the patients (127, 128). Feature selection techniques applied to the characteristics of the targets and the drugs, have allowed advances in the so-called *proteochemometrics*, which aims to optimize the metabolic efficacy of drugs, something that must not be overlooked, in particular when facing polypharmaceutical designs (129).

Machine learning algorithms in cell phenotyping are also starting to gain attention as a route to the design of anti-cancer drugs (130) and repurposing strategies (131). Machine learning in transcriptomic data has been extensively used in recent years, as already discussed. An application that stands out, having revealed the efficacy of a very common over the counter drug (cimetidine, an already off-patent approved anti-ulcer drug with favorable safety profile) to be repurposed to treat lung adenocarcinoma was presented and validated years ago by Sirota and coworkers (132) and its results have been successfully replicated by an independent group (133). The work by Sirota and collaborators exemplifies well one way in which the translational bioinformatics approach should proceed. Starting with high throughput, highly curated information from the CMAP (6), they applied machine learning tools (at that time in the state of the art), discovered novel dysregulated pathways, in lung adenocarcinoma, find key genes involved, look up for FDA approved targets. Validated their findings in cell lines and mouse xenografts and make their data and codes available to allow for replication studies. After this, they started clinical trials to make the treatment available to the patients. If one were to summarize the ‘ideal’ workflow of translational bioinformatics, the work by Sirota and collaborators may be a very good example (132).

Two nascent applications of ML to drug repurposing in cancer are the use of computational learning in electronic health records (EHR) databases (134, 135) and in immune profiles (17, 136). Both are promising for different reasons: On the one hand, EHR databases may provide massive access to data at a relatively low cost, enabling hypothesis generation to be tested in molecular/omic studies. On the other hand, immune ‘fingerprinting’ has shown to be somehow less heterogeneous at the individual level than genomic/transcriptomic profiling while at the same time being highly individual-specific.

The emergence of database resources for repurposing such as RepurposeDB (137) is also worth noticing. Particularly relevant is the fact that computational learning approaches and KDD over such databases have revealed that, aside from purely pharmacological and biochemical features, there are also epidemiological factors influencing the effectiveness of a repurposed drug. Scanning the feature selection spaces allows for innovative treatments within the spectrum of repurposed drugs. Such is the case of the DrugPredict algorithm (138) which, based on molecular and epidemiological data, have been used to repurpose the non-steroidal anti-inflammatory drug Indomethacin for the treatment of chemo-resistant ovarian cancer. Since it has been demonstrated that induced robust cell death in primary patient-derived platinum-sensitive and platinum-resistant ovarian cancer cells.

Computational intelligence techniques combined with systems (particularly network) biology studies constitute relevant lines of research to comprehensively map the interactions pertinent to drug repurposing. The work of the group of Dragici in this regard is worth mentioning (139). This group developed an open source bioinformatic drug repurposing tool called DrugDiseaseNet (https://github.com/azampvd/DrugDiseaseNet). With this tool, the team has managed to reproduce the results of several noteworthy repurposing studies, most notable, the one by (132).

Also, using machine learning combined with network approaches, Tan and collaborators were able to analyze the comprehensive Library of Integrated Network Cell Signatures (LINCS) database (140) to uncover specific druggable targets in glioblastoma (48).

### The Impact on Clinical Outcomes

Ultimately, the success or not of drug repurposing schemes—as in every other therapeutic intervention—must be measured in relation to their impact on clinical outcomes. Of course, there are numerous reports, including data from pre-clinical assays, clinical trials, and observational studies supporting the anti-cancer efficacy of a wide range of repurposed drugs (141). Indeed, one main advantage of repositioned drugs is the fact that, often there are extensive data on pharmacokinetic properties and toxicity available.

However, drug repositioning may require further validation on novel side effects—due, for instance, to different dosage—and other considerations for which clinical trials must be run. The outcome of such studies varies widely. For instance, repurposing of raloxifen (a mineral density enhancer), was validated as an anti-breast cancer therapy in a multicentric study in 180 hospitals in 25 countries and become ultimately FDA-approved as a coadjuvant in breast cancer therapy. Digoxin (a cardiac glycoside) on the other hand, even if quite promising in the experimental stage, bring no survival benefit when compared to conventional platinum-based therapy, and also had significant toxicity and pharmacological interactions (141). That was also the case for the repurposing of Latrepirdine, Ceftriaxone and Topiramate (142). All three drugs were extremely promissory on experimental pre-clinical tests and were relatively well evaluated regarding toxicity and side effects, even at anti-tumor doses, but fail to deliver at the clinical outcome test.

Interestingly, translational bioinformatic approaches have been advanced for the evaluation of clinical outcomes in relation to drug repurposing (143). By performing computational literature mining in databases such as ClinicalTrials.gov and others, it has been posible to pre-evaluate clinical outcomes and focusing repurposing trials on possible *red alerts*. Aside from *positive* clinical outcomes, data mining for adverse events, side effects, and drug-drug interactions, is making possible to sped-up clinical trials for repurposing drugs by standardizing, cataloguing, and processing annotated vocabularies (143, 144). However, standardize, large scale clinical outcome data is not easily available. One alternative that has been proposed (142) is that of online, self-reported patient data (145). This approach has several advantages such as faster data collection, reduced costs, and enhanced patient-engagement, but is still facing challenges related to privacy and systematic curation.

Aside from database reporting and archiving, recent efforts have been made in the use of artificial intelligence (AI) and machine learning for the large scale analysis of clinical outcomes (146). An interesting resource in this regard is the *Clinical Outcome Search Space* (COSS), an AI platform for drug repurposing (147). In spite of these advances, not all the experts agree on the actual efficacy of drug repurposing regarding clinical outcomes.

Tran and Prasad (148), for instance, recall that observational studies alone, may be extremely biased by selection and that this may affect some of the drug repurposing strategies, hence many of the repurposing clinical trials are doomed by design. In order to prevent such biases, randomized controlled trials in large, heterogeneous populations, evaluating *oncological outcomes*, even at the adjuvant level are needed. Such was the case, for instance for the repurposing of metformin as a neo-adjuvant therapy. As of 2020, there are 132 completed, 85 under recruitment, and 32 finishing clinical trials for metformin as an anticancer drug as reported in the ClinicalTrials.gov website (149). In spite of the large samplesize of studies such as the TAXOMET, the STAMPEDE, and the METEOR, and the fact that the drug has been discussed for oncological use for some years, there is no consensus on the real significance regarding clinical outcomes. A striking contrast with this has been the relative success of statins as antineoplastic agents to treat lung cancers (150). However, the very fact that we face such enormous differences in clinical outcomes for repurposed drugs call for optimized means to evaluate *a priori* when a repurposing candidate drug is worth to enter clinical trial stages.

### The Role of Artificial Intelligence and Machine Learning in Drug Repurposing: Challenges and Opportunities

As we have already mentioned, one possible avenue of improvement of drug-repurposing analytics is the use of computational intelligence and machine learning approaches. Such views and methods are particularly relevant to try to cope with the enormous challenges in *interpreting* the vast amounts of heterogeneous experimental and clinical data often present in drug repurposing studies in cancer. The challenge to *make sense* of the data has been approached in several ways. One of such methodologies is *baseline regularization* (BR). Kuang and collaborators introduce BR (151) to analyze EHR data, including drug-prescriptions, physical, and biochemical measurements (lab tests, anthropometrics, etc.). BR make use of statistical relationships to account for changes in the patient’s indicators correlating with the use and dosage of certain drugs of interest. These relationships are then used to identify, assess, or validate drug repurposing candidates.

Deep learning methods such as Deep Neural Networks (DNN), Convolutional Neural Networks (CNN), Support Vector Machines (SVM), and Naive Bayesian analysis (NB), as well as Natural Language Processing (NLP), have also been used to find patterns, useful to predict pharmacological effects, from transcriptomic, genomic, EHR, and bibliographic data (124). A DNN method, for instance, was introduced in a study analyzing perturbation experiments from 678 drugs across several cell lines from the LINCS project (152). ML and DNN have also been used for rational drug discovery, moving on from classic measures such as Quantitative Structure-Activity Relationships (QSAR) to high-throughput, event-based studies for the identification of novel and repurposed drugs (153, 154).

Aside from trying to tackle the complexities of data interpretation in experimental and pre-clinical data, ML approaches have been also developed for the inference and prediction of drug response patterns (155). To do so, MoA data, as well as genomic and transcriptomic databases are being complemented with novel experimental techniques such as those based on single cell assays (these techniques and their use in drug repurposing may be discussed in the next subsection). Computational intelligence techniques are being applied *on tandem*, all along the drug repurposing and development strategies, in the so-called end-to-end (E2E) applications (156). However, powerful these approaches are, we have good reasons to be cautious, even skeptical of them, and as is the case with all clinically-inclined interventions, wait until their effectiveness is proven in controlled, randomized clinical trials.

### Novel Omic Approaches: Single-Cell Sequencing, Structural Genomics, Epigenomics

Technical advances in relation to drug repurposing tools not only consist in the development of computational and bioinfomatic tools to analyze existing experimental data types. Some functional features of biological relevance for drug repurposing are indeed being able to probe only be the use of novel experimental ways to measure biological activity (157). We can mention, for instance, the rapidly developing field of single cell sequencing. Single cell biology has been envisioned as a means to comprehend intra-tumor heterogeneity with greater precision, and with this gained knowledge being able to overcome the diagnostic and therapeutic challenges often posed by such enormous cell-to-cell tumor variability (158). One outstanding example of such tumor heterogeneity is glioblastoma multiforme (GBM). One important component of the essential intractability of advanced stage glioblastoma multiforme is precisely cell-to-cell variability, even within the so-called glioma-imitating cell population. To analyze therapeutic challenges in glioblastoma, Niklasson and coworkers analyzed single cell sorted RNASeq libraries derived from biopsy-captured GBM samples (159) to evaluate mesenchymal states connected to therapy resistance *via* immunomodulatory mechanisms.

To study c-MET inhibitors and their potential role in overcoming drug resistance, Firuzi and collaborators studied spheroid models of pancreatic and stellate cells (160). Single cell proteomic assays confirmed previous sequencing findings regarding the relative effects of repurposed drugs tivantinib, PHA-665752 and crizontinib. Single cell RNASEq and single cell shotgun proteomics have also been used in combination to discern the role of cancer associated fibroblasts in chemoresistance inesophageal adenocarcinoma (161). This study shows that phosphodiesterase 5 inhibitors are able to regulate the activated fibroblasts phenotypes in the benign disease and are promising drugs to enhance response to chemotherapy. Multiscale modeling, including the role that single cell models of ErbB receptor-mediated Ras-MAPK and PI3K/AKT signaling, has been used to study the response to a drug-reposition treatment in prostate adenocarcinoma (162). There single cell sequencing assay data was used to account for subclonal heterogeneity. To evaluate ATRi/BD98 inhibition in cell cycle defects induced by ATR inhibitors in cancer cells, single cell sequencing and single cell gel electrophoresis (COMET) were used by Chory and coworkers (163). These single cell assays allowed the researchers to characterize the MoA of the ATR inhibitors *via* inhibition of ATP-dependent chromatin remodeling complexes SWI/SNF.

Aside from single-cell assays, advances in techniques to probe on structural abnormalities in the genome such as microsatellite instabilities, gene fusions and chromotripsis have revealed clues to the design and repurposing of anticancer drugs. In a recent analysis on the use of gene variants and networks for drug repurposing in colorectal cancer, Irhan and collaborators (164) discussed how to use colorectal cancer biomarkers, such as microsatellite instabilities (MSI), for the repurposing of PIK3CA modulators. Finding molecules such as copanlisib, either alone or in combination with nivolumab as promissory drugs. On a similar line of thought, Fong and To (165) presented the use of immune checkpoint inhibitors as effective therapies for colorectal cancer patients with MSI or mismatch repair variants. This has led to the FDA approval of pembrolizumab (Keytruda) combined with nivolumab as PD-1 inhibitors and of ipilimumab as a CLT4-inhibitors in those tumors. In connection to anti-breast cancer therapies, pembrolizumab has also been approved for metastatic tumors with marked MSI. Such is also the case of coadjuvant theory with aspirin and Celecoxib as (anti-PD-1 antibody) for advanced stage breast cancer (166, 167). MSI has also been a factor to consider for the repurposing of co-adjuvant drugs to treat advanced stage melanoma (Indoximod), metastatic non-small cell lung cancer (Metformin), in both cases to enhance pembrolizumab activity.

Another set of structural variants of interest for anti-cancer drug repurposing is that of gene fusions. Perhaps the paradigmatic case is that of acute myeloid leukemia (AML) (168). The case of niclosamide is relevant since it targets some relatively common gene fusions (or their associated chimeric proteins), aside from targeting relevant transcription factors such as CREB, STAT3 and NF-*κ*B. Chromosomal aberrations and gene fusions in intimal sarcoma have also helped to identify potential therapeutic targets (169). In particular, the PDE4NIP/NOTCH2 and the MRPS30/ARD2 fusion positive tumors have been identified as druggable targets. In colon cancer, KCTD12/CDK1 fusion positive tumors have been shown to become vulnerable to vemurafenib *via* a coadjuvant treatment with adefovir dipivoxil (170). This allows the repurposing of the BRAF V600E inhibitor vemurafenib from melanoma to colon cancer therapy.

Epigenomic markers—most notably methylation patterns—have also unveiled avenues for drug repurposing (171). Some of these were found *via* KsRepo a methylation-based drug repurposing method for acute myeloid leukemia (172) that has allowed to reposition four drugs: alitretinoin, cytarabine, panabinostat, and progesterone for AML. Methylation profiles (in particular, m^6^A DNA/RNA methylation) have been proven to be relevant to the action of repurposed drugs such as afatinib in non-small cell lung cancer (173). DNA methylation profiles also have been useful as a tool to find out novel and repurposed therapeutic targets in bladder cancer (174).

In particular, a novel useof 5-azacytidine a nucleoside analogue and decitabine that may function as a DNA methyltransferase inhibitor, have been found to re-activate tumor suppressor genes, inhibiting tumor cell growth and increasing apoptosis in bladder cancer cells. These results remain consistent from *in vitro* assays all along to clinical trials.

### Drug Delivery Mechanisms and Chemo-Resistance

A relevant and often overlooked challenge in drug repurposing—in particular when the repositioned drug was originally a non-oncological one—is the issue of drug delivery efficacy and its relationship with proper drug targeting and chemo-resistance. One example of how to overcome these challenges is the reduction of chemo-resistance *via* coadjuvant therapy with mebendazole (175). Aside from coadjuvant therapy, perhaps the best solution to optimize drug delivery to the tumors is *via* advancing delivery technologies (176, 177). Lei and coworkers, for instance, discussed the use of nanomedicine such as nanoparticle albumin-bound paclitaxel (nab-PTX), abraxane, or a liposomal formulation of irinotecan as effective improvements of anti-cancer drug delivery for pancreatic ductal adenocarcinoma (177).

The use of exosomes has been extensively studied recently, in particular since they may play a role, not only in drug delivery, but also in regulating autocrine and paracrine signaling pathways which may regulate drug responses (178). Extracellular vesicles have also been used to navigate through the tumor microenvironment in glioblastoma. Such vesicles have resulted useful to deliver drugs, even through the blood-brain barrier (179). Caution, however, mut be taken since these vesicles have also biological roles such as the promotion of angiogenesis, immune suppression and facilitating recurrence, all of them pro-tumor effects. Hence, a lot of research efforts must be devoted to develop effective drug delivery mechanisms that enhance drug-targeting and reduce chemo-resistance in relation to anti-cancer repurposed therapy.

### Emerging Proteome-Based Studies

We have mentioned that most high-throughput pathway activity and drug MoA studies are based on either sequencing known genomic targets or novel mutations or measuring gene expression by RNASeq, microarrays, or Luminex-type assays. However, quite recently proteomic-wise techniques are enhancing our capacities to probe cellular activity at the (functional) proteome and phospho-proteome level. One of such techniques is isobaric labeling mass spectrometry (8). This technique has allowed to identify and dose-stratify the binding of the drug staurosphorine to 228 cellular kinases on a single experiment. Proteomic and phosphoproteomic analyses have also allowed to reveal mechanisms of activation of NEK2 and AURKA kinases in cancer (180), thus allowing the use of drugs targeting such kinases in six different cancer types within the Clinical Proteomic Tumor Analysis Consortium (CPTAC): Breast cancer, clear cell renal carcinoma, colon cancer, lung adenocarcinoma, ovarian cancer, and uterine corpus endometrial carcinoma.

Advances in the experimental tools to study cancer proteome-wise, have also called for the development of new methodological, computational, and analytical techniques, useful in drug repurposing strategies. As an example, Saei and collaborators developed a comprehensive chemical proteomics profiling approach for target deconvolution of a redox active drug auranofin (originally and anti-rheumatic called Ridaura) as an anti-cancer drug auranofin was found to target genes such as TXNRD1, NFKB2, and CHORDC1, all of them known to be involved in the perturbation of oxidoreductase pathways in cancer (181). Bioinformatic platforms for the predictive analytics of drug-protein-disease data are in turn, being developed. Such is the case of rb”cando.py”, a bioinformatic platform to analyze changes in proteome profiles related to drug perturbation. This method has been applied successfully to analyze repurposing of ribavririn and a novel compound LMK-235 in breast cancer and AML. The results have been validated in *in vivo* experiments and are being considered to enter a clinical phase in the near future (182). These are but a handful of examples that, however, make us anticipate further, near-future developments, in the high-throughput study of phenomena of interest for systematic drug repositioning strategies to treat cancer.

### Concluding Remarks

Drug repurposing in cancer is a quite complex endeavor. In order to cope with all the complexities and subtleties involved, there is a need for collaborative, multidisciplinary teams, including clinical oncologists and oncological surgeons, molecular oncologists, cancer cell biologists and pharmacologists but also bioinformaticians, computational biologists and data scientists. One emergent and quite successful avenue of research and intervention, is that of basing repurposing strategies on functional, semi-mechanistic basis as the one supplied by pathway-based analysis. This comes as no surprise, since the ultimate goal of pharmacological interventions is the modulation of functional traits and processes both at the functional and physiological levels. Hence pathway-based studies provide a close proxy as to these functional processes that make us hypothesize that findings based on these may prove to be more effective in terms of providing effective anticancer therapy.

The present review discusses recent advances in the application of computational molecular biology and bioinformatic approaches using high throughput omic data, mining of extensive, well-annotated databases and a cycle of experimental and clinical validation, to face some of the more evident challenges for anti-cancer drug repurposing. The field is flourishing so this review is not meant to be comprehensive but rather to serve as an introductory journey into a wide and fascinating research topic.

## Author Contributions

EH-L devised the project, established the outline, and made the figures. EH-L and MM-G performed literature surveys and revisions. EH-L and MM-G wrote the manuscript. All authors contributed to the article and approved the submitted version.

## Funding

This work was supported by the Consejo Nacional de Ciencia y Tecnología (SEP-CONACYT-2016-285544 and FRONTERAS-2017-2115), and the National Institute of Genomic Medicine, México. Additional support has been granted by the Laboratorio Nacional de Ciencias de la Complejidad, from the Universidad Nacional Autónoma de México. EH-L is recipient of the 2016 Marcos Moshinsky Fellowship in the Physical Sciences.

## Conflict of Interest

The authors declare that the research was conducted in the absence of any commercial or financial relationships that could be construed as a potential conflict of interest.
